# Retinal degeneration modulates intracellular localization of CDC42 in photoreceptors

**Published:** 2011-11-15

**Authors:** S.R. Heynen, N. Tanimoto, S. Joly, M.W. Seeliger, M. Samardzija, C. Grimm

**Affiliations:** 1 Laboratory for Retinal Cell Biology, Department of Ophthalmology, University of Zurich, Switzerland; 2 Division of Ocular Neurodegeneration, Centre for Ophthalmology, Institute for Ophthalmic Research, University of Tuebingen, Germany; 3 Zurich Center for Integrative Human Physiology, University of Zurich, Switzerland; 4 Center for Neuroscience Zurich (ZNZ), University of Zurich, Switzerland

## Abstract

**Purpose:**

Rho GTPases such as RAS-related C3 botulinum substrate 1 (RAC1) and cell division cycle 42 homolog (*S. cerevisiae*; CDC42) have been linked to cellular processes including movement, development, and apoptosis. Recently, RAC1 has been shown to be a pro-apoptotic factor in the retina during light-induced photoreceptor degeneration. Here, we analyzed the role of CDC42 in the degenerating retina.

**Methods:**

Photoreceptor degeneration was studied in a mouse model for autosomal dominant retinitis pigmentosa (VPP) with or without a rod-specific knockdown of *Cdc42*, as well as in wild-type and *Cdc42* knockdown mice after light exposure. Gene and protein expression were analyzed by real-time PCR, western blotting, and immunofluorescence. Retinal morphology and function were assessed by light microscopy and electroretinography, respectively.

**Results:**

CDC42 accumulated in the perinuclear region of terminal deoxynucleotidyl transferase dUTP nick end labeling–negative photoreceptors during retinal degeneration induced by excessive light exposure and in the *rd1*, *rd10*, and VPP mouse models of retinitis pigmentosa. The knockdown of *Cdc42* did not affect retinal morphology or function in the adult mice and did not influence photoreceptor apoptosis or molecular signaling during induced and inherited retinal degeneration.

**Conclusions:**

Retinal degeneration induces the accumulation of CDC42 in the perinuclear region of photoreceptors. In contrast to RAC1, however, lack of CDC42 does not affect the progression of degeneration. CDC42 is also dispensable for normal morphology and function of adult rod photoreceptor cells.

Received: May 25, 2011

Accepted: November 10, 2011

## Introduction

Retinitis pigmentosa (RP) and age-related macular degeneration are diseases that result in the loss of vision due to photoreceptor apoptosis [1,2]. To study mechanisms of photoreceptor death, several mouse models of RP have been developed. Exposure to white light is an inducible model in which the severity of degeneration depends on light intensity and duration of exposure [3]. In this model, photoreceptors die and are cleared from the subretinal space within a period of approximately 10 days. Mouse models of inherited retinal degeneration include retinal degeneration (*rd*)1 [4], *rd10* [5], VPP [6], and others [7]. *Rd1* and *rd10* mice carry a recessive nonsense or missense mutation, respectively, in the β-subunit of the cGMP phosphodiesterase gene. In *rd1*, this results in an early onset (postnatal day [P]10) and rapid photoreceptor degeneration, whereas in *rd10* the degeneration has a later onset (P15) and a slower progression. The VPP mouse expresses a rhodopsin transgene encoding a mutant protein with three amino acid substitutions (V20G, P23H, P27L). Photoreceptor cell death in this mouse begins around P15 and progresses over several weeks.

Rho guanosine triphosphate (GTP)ases such as RAS-related C3 botulinum substrate 1 (*Rac1*) and cell division cycle 42 homolog (*S. cerevisiae*; *Cdc42*) are well known modulators of microtubule and actin structures [8]. Rho GTPases cycle between an inactive guanosine diphosphate–bound state and an active GTP-bound state [9]. Active Rho GTPases bind to a host of different effector proteins [10-13] to elicit a myriad of signaling responses involved in the regulation of cellular movement, adhesion, axon guidance, differentiation, and apoptosis [13-17]. Despite the importance of Rho GTPases in many physiologic and pathophysiological processes, only little is known about their roles in the eye.

Although few in number, there have been some studies on CDC42 documenting a variety of ocular functions. For example, CDC42 has been shown to be important for wound-healing processes in the corneal endothelium [18]. In addition, CDC42 was suggested to be involved in lens pit invagination during eye morphogenesis [19] and—based on the spatial and temporal expression pattern—in retinal development [20]. Despite these studies, there is a lack of understanding of the function of CDC42 in the mature retina. RAC1, however, has recently been implicated in photoreceptor degeneration as a pro-apoptotic factor by Haruta and colleagues [21], and is thus an interesting target for therapeutic interventions. Since RAC1 and CDC42 are members of the same family of proteins, and since they can have overlapping functions [12,22], we addressed the question of whether CDC42—similar to RAC1—might also influence processes involved in retinal degeneration. To be able to directly compare the results obtained for CDC42 to the RAC1 data published recently [21], we used the same experimental approach as published and analyzed retinal degeneration in mice, specifically in rod photoreceptors, with a conditional *Cdc42* knockdown.

## Methods

### Animals and light exposure

All procedures were conducted in accordance with the guidelines published by the Institute for Laboratory Animal Research and with the regulations of the Veterinary Authority of Zurich. *Cdc42* floxed mice (*Cdc42^flox/flox^*) [23] were a generous gift from Dr. Joao Relvas (Institute of Cell Biology, ETH Zurich, Zurich, Switzerland). The 129S6/SvEvTac mice were purchased from Taconics (Eiby, Denmark). Mice with Cre recombinase expression controlled by the rod opsin promoter (LMOPC1, which will be called opsin-Cre from now on) [24] were provided by Dr. Yun Le (University of Oklahoma, Oklahoma City, OK). The mouse model for autosomal dominant RP (called VPP from now on) was kindly provided by Dr. Muna Naash (University of Oklahoma). Pde6brd10 [4] (called *Rd10* from now on) and Pde6brd1 [5] (called *Rd1* from now on) were purchased from Jackson laboratories (Bar Harbor, MA) and Harlan (Horst, Netherlands), respectively. A summary of mouse models for inherited retinal degeneration used in this study is given in Table 1. *Rd1* and *rd10* mice were homozygous for the *Rpe65_450Met_* variant, whereas all other strains were homozygous for *Rpe65_450Leu_* [25]. All mice were kept as breeding colonies at the animal facility of the University Hospital Zurich in a 12 h:12 h light-dark cycle with 60 lux at cage level. All experiments were performed with 12-week-old mice unless stated otherwise.

**Table 1:** Mouse models of inherited retinal degeneration used in this study

**Animal model**	**Gene**	**Mutation / Transgene**	**Time course of degeneration**	**Model for disease**	**Origin**	**Reference**
Rd1	*Pde6b*	Nonsense	PND8–21	arRP	Harlan, Horst, The Netherlands	[4]
Rd10	*Pde6b*	Missense	PND16–60	arRP	Jackson Laboratory, Bar Harbor, ME	[5]
VPP	*Rho*	Tg(V20G,P23H,P27L)	PND20–250	adRP	Dr. Muna Naash	[6]

Abbreviations: *Pde6β*: Phosphodiesterase 6β; *Rho*: Rhodopsin; adRP: autosomal dominant retinitis pigmentosa, arRP: autosomal recessive retinitis pigmentosa, Rd: retinal degeneration, PND: postnatal day, Tg: transgene.

For light exposure experiments, mice were dark adapted overnight and pupils were dilated with 1% cyclopentolate (Cyclogyl; Alcon, Cham, Switzerland) and 5% phenylephrine (Ciba Vision, Niederwangen, Switzerland) 30 min before exposure to 13,000 lux of white light for 2 h, as described previously [26].

### Generation of rod photoreceptor–specific Cdc42 knockdown mice and genotyping

For the experiments, *Cdc42^flox/flox^*;*opsin-Cre* mice were crossed with *Cdc42^flox/flox^* mice to obtain litters with *Cdc42^flox/flox^;opsin-Cre* (rod photoreceptor–specific *Cdc42* knockdown) and *Cdc42^flox/flox^* (control mice). The late onset of Cre recombinase expression postnatally allowed a normal retinal development, permitting the use of the *Cdc42^flox/flox^*;*opsin-Cre* to study the role of CDC42 in adult rod photoreceptor cells.

Genotypes were determined using genomic DNA from ear biopsies, and the following conventional PCR conditions: initial denaturation (95 °C, 5 min); 35 cycles of denaturation (95 °C, 45 s), annealing (60 °C, 45 s), and elongation (72 °C, 45 s); and final extension (72 °C, 10 min). For the detection of the floxed *Cdc42* allele, primers (forward: 5′-TTC TTC CTC CAA CCT CCT GAT GGG-3′, reverse 5′-TGC TGT GTG TGG CAT TTG CTG C-3′) spanning both *loxP* sites were used. The amplicon of the floxed allele was 1.5 kb long, whereas amplification of the wild-type allele yielded a 1.3 kb fragment. Excision of the floxed sequence by the Cre recombinase resulted in an amplification product of 0.9 kb. For the detection of the *Cre* transgene, primers (forward: 5′-AGG TGT AGA GAA GGC ACT TAG C-3′, reverse 5′-CTA ATC GCC ATC TTC CAG CAG G-3′) specific for *Cre* were used. PCR products were run on a 1% agarose gel for size detection.

### RNA preparation and semiquantitative RT–PCR

Retinas were removed through a slit in the cornea and immediately frozen in liquid nitrogen. Total RNA was prepared with an RNA isolation kit (RNeasy; Qiagen, Hilden, Germany) including a DNase treatment to remove residual genomic DNA. Identical amounts of RNA were used for reverse transcription using oligo(dT) and M-MLV reverse transcriptase (Promega, Madison, WI). Gene expression was analyzed by real-time PCR using specific primer pairs (Table 2) spanning an intronic region of the respective gene, a polymerase ready mix (LightCycler 480 SYBR Green I Master Mix; Roche Diagnostics, Indianapolis, IN), and a thermocycler (LightCycler Roche Diagnostics). Signals were normalized to *Actb* and relative expression was calculated by the comparative threshold cycle (ΔΔCT) method using a control sample for calibration [27].

**Table 2:** Primers and conditions for real time PCR

	**Oligonucleotide primers**			
**Gene**	Forward 5′-3′	Reverse 5′-3′	**Annealing temperature (°C)**	**Product (bp)**
*Cdc42*	GGCGGAGAAGCTGAGGACAAG	AGCGGTCGTAGTCTGTCATAATCCTC	60	275
*Gnat1*	GAGGATGCTGAGAAGGATGC	TGAATGTTGAGCGTGGTCAT	58	209
*Chx10*	CCAGAAGACAGGATACAGGTG	GGCTCCATAGAGACCATACT	60	111
*Opn4*	CCAGCTTCACAACCAGTCCT	CAGCCTGATGTGCAGATGTC	62	111
*Actb*	CAACGGCTCCGGCATGTGC	CTCTTGCTCTGGGCCTCG	62	153
*Rho*	CTTCACCTGGATCATGGCGTT	TTCGTTGTTGACCTCAGGCTTG	62	130
*Opn1sw*	TGTACATGGTCAACAATCGGA	ACACCATCTCCAGAATGCAAG	58	153
*Opn1mw*	CTCTGCTACCTCCAAGTGTGG	AAGTATAGGGTCCCCAGCAGA	58	154
*Lif*	AATGCCACCTGTGCCATACG	CAACTTGGTCTTCTCTGTCCCG	60	216
*Edn2*	AGACCTCCTCCGAAAGCTG	CTGGCTGTAGCTGGCAAAG	60	64
*Fgf2*	TGTGTCTATCAAGGGAGTGTGTGC	ACCAACTGGAGTATTTCCGTGACCG	62	158
*Gfap*	CCACCAAACTGGCTGATGTCTAC	TTCTCTCCAAATCCACACGAGC	62	240
*Stat3*	CAAAACCCTCAAGAGCCAAGG	TCACTCACAATGCTTCTCCGC	62	159
*Stat1*	TTGTGTTGAATCCCGAACCT	TCGAACCACTGTGACATCCT	62	95
*Socs3*	ATTTCGCTTCGGGACTAGC	AACTTGCTGTGGGTGACCAT	58	126

### Laser capture microdissection

Mouse eyes were enucleated and immediately frozen in tissue-freezing medium using a methylbutanol bath cooled by liquid nitrogen. Retinal sections (20 µm) were cut, fixed (5 min acetone), air dried (5 min), and dehydrated (30 s in 100% ethanol, 5 min in xylol). Microdissection of the retinal nuclear layers was performed with an Arcturus XT Lasercapture device (Molecular Devices, Silicon Valley, CA). RNA was isolated using the Arcturus RNA isolation kit (Molecular Devices), and residual genomic DNA removed by a DNase treatment. cDNA synthesis was performed as described in the previous section. Gene expression was determined by conventional PCR (40 cycles) or semiquantitative PCR using the primer pairs listed in Table 2. Amplified fragments were run on a nondenaturing polyacrylamide gel and detected after staining with ethidium bromide.

### Light microscopy and spider diagram

Enucleated eyes were fixed in 2.5% glutaraldehyde in 0.1 M cacodylate buffer (pH 7.3) overnight at 4 °C. The cornea and lens were removed and the superior and inferior retina of each eye was prepared, washed in cacodylate buffer, incubated in osmium tetroxide for 1 h, dehydrated in a series of increasing ethanol concentrations, and embedded in Epon 812. Semithin cross-sections (500 nm) were prepared from the ventral central retina, the most affected region in our light-damage model [3]. Sections were counterstained with toluidine blue and analyzed by light microscopy.

The thickness of the photoreceptor nuclear layer was measured using the Adobe Photoshop CS3 ruler tool at 250, 500, 1,000, 1,500, 1,750, 2,000, and 2,250 μm distances from the optic nerve head in both the dorsal and ventral directions. Results of n=3 retinas were plotted as a spider diagram.

### Immunofluorescence

Eyes were enucleated and fixed in 4% (wt/vol) paraformaldehyde in 0.1 M phosphate buffered saline (PBS; pH 7.4) overnight. After removing the cornea and lens, eyecups were postfixed in 4% paraformaldehyde for an additional 2 h before being immersed in 30% sucrose in PBS at 4 °C overnight. The eyes were embedded in tissue-freezing medium (Leica Microsystems Nussloch GmbH, Nussloch, Germany) and frozen in a 2-methylbutane bath cooled by liquid nitrogen. Retinal sections (12 µm) were cut, placed on slides and incubated with a blocking solution (3% normal goat serum, 0.3% Triton X-100 in 0.1M PBS, or 3% horse serum, 0.3% Triton X-100 in 0.1M PBS) for 1 h at room temperature. For protein detection, sections were incubated with primary antibodies (Table 3) diluted in blocking solution at 4 °C overnight. After three washes with PBS, slides were incubated with the appropriate secondary antibody coupled to Cy3 or Cy2 for 1 h at room temperature, washed, counterstained with 4',6-diamidino-2-phenylindole (DAPI), and mounted with antifade medium (10% Mowiol 4–88; vol/vol; Calbiochem, San Diego, CA), in 100 mM Tris (pH 8.5), 25% glycerol (wt/vol), and 0.1% 1,4-diazabicyclo (2.2.2) octane.

**Table 3:** Antibodies for immunoblotting and immunofluorescence

**Antigen**	**Host**	**Dilution**	**Catalog number**	**Company**
CDC42	Rabbit	1:500	Sc-87	Santa Cruz Biotechnology
RHO	Mouse	1:100	-	Gift from Dr. Hicks
GNAT1	Rabbit	1:500	Sc-389	Santa Cruz Biotechnology
OPN1SW	Goat	1:500	Sc-14363	Santa Cruz Biotechnology
JAK2	Rabbit	1:500	#44–604	Invitrogen, Basel, Switzerland
p-JAK2	Rabbit	1:250	#44–426	Invitrogen
STAT1	Rabbit	1:1000	#9172	Cell signaling technology, Beverly MA
p-STAT1	Rabbit	1:1000	#9171	Cell signaling technology
STAT3	Rabbit	1:1000	#9132	Cell signaling technology
p-STAT3	Rabbit	1:500	#9131	Cell signaling technology
AKT	Rabbit	1:2500	#9272	Cell signaling technology
p-AKT	Rabbit	1:1000	#9271	Cell signaling technology
ACTB	Mouse	1:5000	#5441	Sigma, St. Louis, MO

### TUNEL assay and cell death detection

Detection of photoreceptor cell death in retinal sections was done using the terminal deoxynucleotidyl transferase dUTP nick end labeling (TUNEL) method according to the manufacturer’s recommendations (Roche Diagnostics, Rotkreuz, Switzerland). Immunofluorescent signals were analyzed with a digital microscope (Axiovision; Carl Zeiss Meditec, Inc., Dublin, CA).

Apoptotic cell death was quantified 36 h after light exposure by measuring the release of free nucleosomes in isolated retinas using the cell death detection kit (Roche Diagnostic, Basel, Switzerland) according to the manufacturer’s instructions. Briefly, retinas were homogenized and the free nucleosomal content was quantified photometrically by a sandwich-enzyme-immunoassay principle using a spectrophotometer.

### Western blot analysis

Retinas were homogenized in 0.1 M Tris/HCl (pH 8.0) and protein content was analyzed using Bradford reagent. Equivalent amounts of proteins were resolved by electrophoresis on sodium dodecyl sulfate–polyacrylamide gels and transferred to nitrocellulose membranes. Membranes were blocked in 5% milk (Bio-Rad, Hercules, CA) in TBST (10 mM Tris/HCl [pH 8.0], 150 mM NaCl, and 0.05% Tween-20) for 1 h at room temperature before they were incubated overnight at 4 °C in 5% milk (in TBST) containing the respective primary antibody (Table 3). Detection was with horseradish peroxidase (HRP)-conjugated secondary antibodies and proteins were visualized using the Renaissance Western Blot Detection Kit (PerkinElmer Life Sciences, Boston, MA).

### Electroretinography

Electroretinograms (ERGs) were recorded from both eyes simultaneously, as previously described [28,29]. Briefly, mice were dark-adapted overnight and anesthetized the next day with ketamine (66.7 mg/kg) and xylazine (11.7 mg/kg). Pupils were dilated before performing single flash ERG recordings under dark-adapted (scotopic) followed by light-adapted (photopic) conditions. Light adaptation was accomplished with a background illumination of 30 cd/m^2^ starting 10 min before photopic recording. Single white-flash stimulus intensity ranged from –4 to 1.5 log cd*s/m^2^ under scotopic and from –2 to 1.5 log cd*s/m^2^ under photopic conditions, divided into 10 and 8 steps, respectively. Ten responses were averaged with an interstimulus interval of either 5 s or 17 s (for 0, 0.5, 1, and 1.5 log cd*s/m^2^).

### Statistical analysis

Statistical analyses were performed using Prism4 software. All data are the mean ± standard deviation (SD) of three animals per group. Statistical differences of means were calculated using ANOVA (ANOVA) followed by a Bonferroni post-hoc test. A p value of less than 0.05 was considered significant.

## Results

### Accumulation of CDC42 in the perinuclear region of TUNEL–negative photoreceptors during retinal degeneration

**Figure 1:**
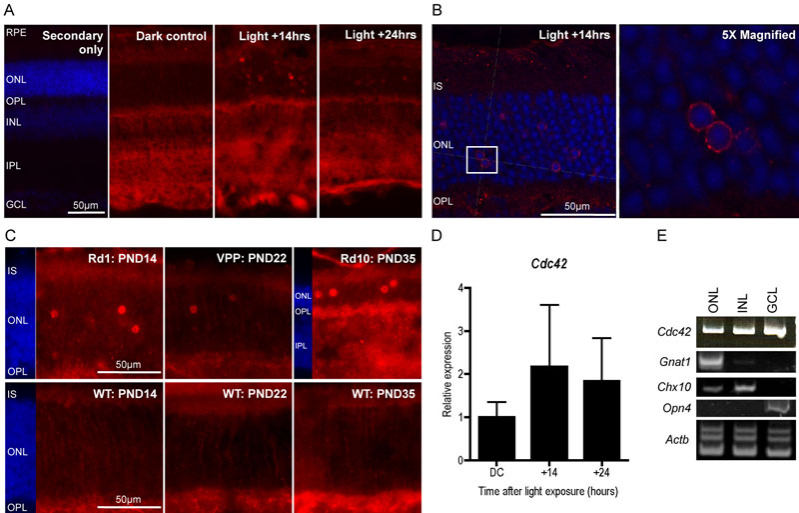
Cell division cycle 42 homolog (S.cerevisiae; CDC42) localizes to the perinuclear region of photoreceptors during retinal degeneration. **A**: CDC42 was immunofluorescently labeled in retinal sections of dark control mice and in mice at 14 h and 24 h after light exposure. The first panel shows the control of a light exposed retina (at 14 h after exposure) stained with the secondary antibody alone. Shown are representative stainings of n=3 mice. **B**: The outer nuclear layer (ONL) of CDC42 stained retinal sections of mice at 14 h after light exposure, were analyzed by confocal microscopy. The boxed area is shown at higher magnification in the right panel. **C**: Retinal sections of retinal degeneration (*rd*)1, *rd10*, autosomal dominant retinitis pigmentosa (VPP) and wild-type age matched controls at indicated post-natal days were immunofluorescently stained for CDC42. **D**: Relative gene expression of *Cdc42* was analyzed in retinas of dark controls (DC) and in retinas at 14 h and 24 h after light exposure. Shown are mean values±SD of 3 independent mice. **E**: Retinal layers were isolated by laser capture microdissection and examined for *Cdc42* expression. *Gnat1* (ONL), *Chx10* (inner nuclear layer; INL), *Opn4* (ganglion cell layer; GCL) served as controls to assess purity of isolated layers and Actb was amplified as a loading control. Blue: nuclei (4’,6 diamidino-2-phenylindole [DAPI] staining). Red: CDC42. Scale bars 50 μm. RPE: retinal pigment epithelium, IPL: inner plexiform layer, IS: photoreceptor inner segment, PND: post-natal day.

Immunofluorescent stainings of CDC42 on retinal sections showed that CDC42 was ubiquitously expressed in the retina. This was supported by analysis of *Cdc42* mRNA (mRNA) expression in individual retinal layers separated by laser capture microdissection (Figure 1E). Localization of CDC42 in the inner retinal layers remained unchanged after light exposure (Figure 1A) and in the genetic models of photoreceptor degeneration studied (not shown). However, after induction of photoreceptor degeneration by light, CDC42 was additionally detected in distinct cell bodies in the outer nuclear layer (ONL; Figure 1A). Closer inspection of CDC42 localization by confocal microscopy revealed that CDC42 accumulated in the perinuclear region of photoreceptors (Figure 1B). Perinuclear accumulation of CDC42 in the ONL of degenerating retinas was an early and transient event, peaking around 14 h after light exposure. At this time point, *Cdc42* showed a tendency of increased gene expression (Figure 1D). A similar CDC42 staining pattern was observed in all tested inherited mouse models (*rd1*, *rd10*, and VPP) of retinal degeneration (Figure 1C). This suggested that the perinuclear accumulation of CDC42 in individual cells of the ONL is a common mechanism in retinas with degenerating photoreceptors, and is independent of the toxic stimulus (light, mutation) that induced cell death.

**Figure 2:**
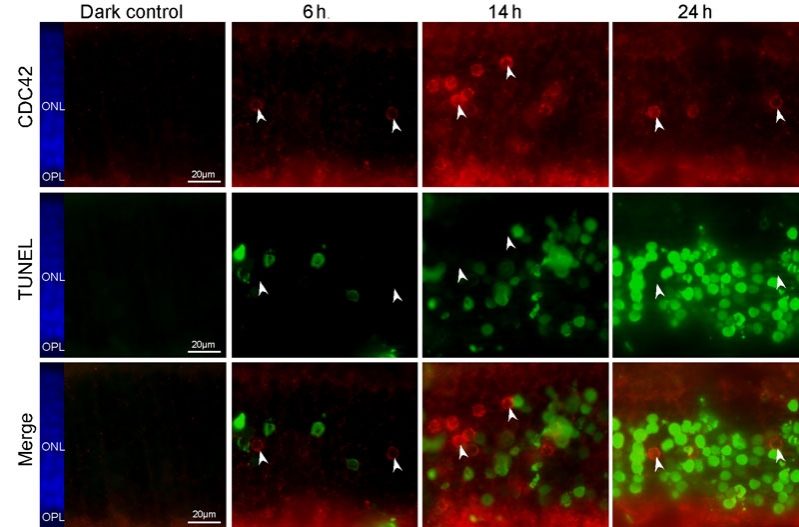
Cell division cycle 42 homolog (S.cerevisiae; CDC42) accumulates in terminal deoxynucleotidyl transferase dUTP nick end labeling (TUNEL)-negative photoreceptors after light exposure. Retinal sections of light exposed wild type mice were co-immunostained for CDC42 and TUNEL. Time points after light exposure are indicated and arrowheads highlight CDC42 positive cells. Red: CDC42. Green: TUNEL. Blue: nuclei (4’,6 diamidino-2-phenylindole [DAPI] staining) Scale bar: 20 μm. ONL: outer nuclear layer.

Costaining for CDC42 and apoptotic cells using the TUNEL assay showed that perinuclear CDC42 staining appeared around 6 h after light exposure and was strongest around 14 h (Figure 2). It decreased rapidly thereafter as the number of TUNEL-positive cells rose (Figure 2). At the peak of apoptosis, around 36 h after light exposure [3], perinuclear CDC42 staining was no longer detectable in photoreceptors (data not shown). Importantly, CDC42 accumulated in the perinuclear region exclusively in TUNEL-negative photoreceptors, suggesting that CDC42 may support survival of cells or, alternatively, may prepare photoreceptors to die.

### Rod photoreceptor–specific knockdown of Cdc42

**Figure 3:**
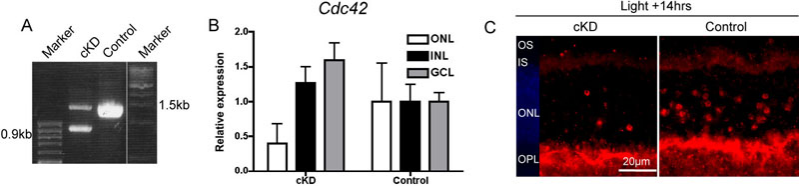
Cell division cycle 42 homolog (S.cerevisiae; *Cdc42*) conditional knockdown was achieved specifically in rod photoreceptor cells. **A**: PCR products after amplification of the floxed region of *Cdc42* from retinal genomic DNA of *Cdc42* conditional knockdown (cKD) and control mice at 12-weeks of age were separated and visualized by DNA agarose electrophoresis. Amplification of the floxed sequence results in a 1.5 kb fragment and of the excised sequence in a 0.9 kb product. **B**: Relative expression of *Cdc42* in the outer nuclear layer (ONL; white box), the inner nuclear layer (INL; black box), and the ganglion cell layer (GCL; gray box) of *Cdc42* knockdown and control mice was analyzed by real-time PCR after laser capture microdissection. Shown are mean values±SD of 3 independent mice. Expression of *Cdc42* in each retinal layer of control mice was set to ‘1’. **C**: Retinal sections from *Cdc42* knockdown and control mice 14 h after light damage were immunofluorescently stained for CDC42. Images are representatives of 3 independent mice per genotype. Red: CDC42. Blue: nuclei (4’,6 diamidino-2-phenylindole [DAPI] staining). Scale: 20 μm. OS: photoreceptor outer segments. IS: photoreceptor inner segments.

To address the role of CDC42 in photoreceptor survival or death directly, we generated *Cdc42^flox/flox^;opsin-Cre* mice that lack functional CDC42 specifically in rods. Although the expression of Cre recombinase has been described as being somewhat patchy in opsin-cre mice, expression is specific to rods and affects about 75% of photoreceptors [24,30]. By 12 weeks of age, Cre-mediated excision of *Cdc42* genomic sequences was analyzed by amplifying genomic DNA isolated from retinal tissue. A 0.9 kb fragment was detected in *Cdc42* knockdown (*Cdc42^flox/flox^;*opsin-Cre) but not in control *Cdc42^flox/flox^* mice, indicating successful excision of the floxed exon 2 of *Cdc42* in the presence of Cre recombinase (Figure 3A). Since Cre recombinase was specifically expressed in rods, other retinal cells still carried the full-length floxed *Cdc42* gene. Thus, PCR amplification of total retinal genomic DNA resulted in two bands representing the knockdown (0.9 kb) and floxed exon 2 (1.5 kb) alleles of *Cdc42*, respectively. A successful knockdown of *Cdc42* in the ONL was also determined by the relative quantification of *Cdc42* mRNA levels in the individual retinal layers after laser capture microdissection. Expression of *Cdc42* was reduced by 60% in the ONL of *Cdc42* knockdown mice relative to control mice (Figure 3B). Thus, we estimate that about 60% of the floxed *Cdc42* alleles have been deleted in the ONL. Given that only about roughly 75% of rods express the CRE recombinase [24] we expect that 25% to 30% of rods are wild type for *Cdc42* (*Cdc42^+/+^*). Of the remaining rods, 40% to 50% have to be full knockouts (*Cdc42^−/−^*), whereas the remaining 20% to 30% may be *Cdc42^+/−^*. The reason for the slightly elevated levels of *Cdc42* mRNA in the inner nuclear layer (INL) and the ganglion cell layer (GCL) of knockdown mice is not known, but may potentially include some reactions compensating for the absence of *Cdc42* in the ONL. The reduced expression of *Cdc42* in the ONL of knockdown mice was also reflected in a reduced number of photoreceptors with a perinuclear localization of CDC42 in the degenerating retina (Figure 3C). Together, these results suggested an effective CDC42 knockdown at 12 weeks of age. All subsequent experiments were performed on animals at this age, unless otherwise stated.

### Rod-specific ablation of CDC42 does not affect retinal morphology and function

**Figure 4:**
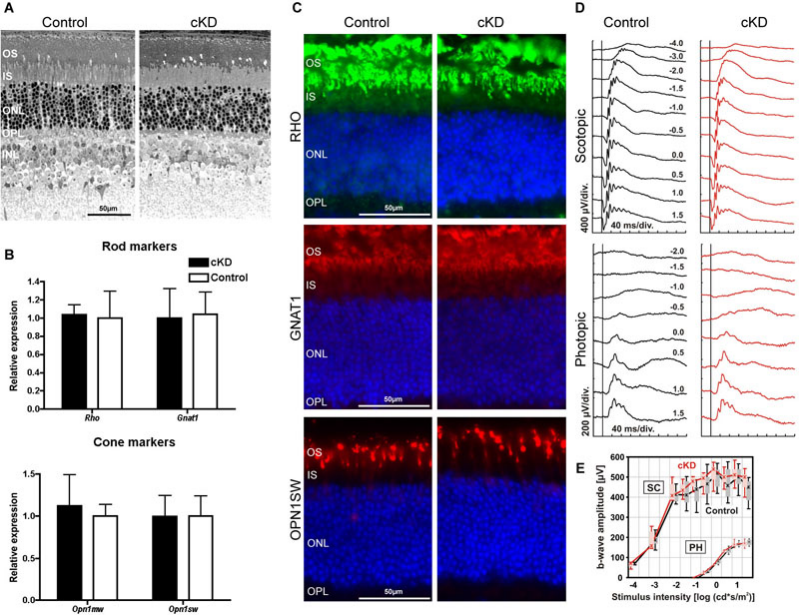
Ablation of cell division cycle 42 homolog (S.cerevisiae; CDC42) does not affect retinal morphology and function. **A**: The retinal morphology of *Cdc42* conditional knockdown (cKD) and control mice was examined. Shown are representative sections of 3 independent animals per genotype. **B**: Gene expression levels of rod (rhodopsin, *Rho*; rod transducin, *Gnat1*) and cone markers (middle wavelength cone opsin, *Opn1mw*; short wavelength cone opsin, *Opn1sw*) were analyzed in 12-week-old *Cdc42* knockdown (black box) and control mice (white box). Shown are mean values±SD, of 3 independent mice. Expression in control mice was set to ‘1’. **C**: Retinal sections of *Cdc42* knockdown and control mice were stained for RHO (top panels, green), GNAT1 (middle panels, red), and OPN1sw (bottom panels, red). Blue: nuclei (4’,6 diamidino-2-phenylindole [DAPI] staining). Shown are representative sections from 3 mice. **D**: Representative scotopic (dark-adapted) and photopic (light-adapted) single flash electroretinogram (ERG) recordings with increasing light intensities show retinal function of *Cdc42* knockdown (red line) and control (black line) mice at 12 weeks of age. The vertical line shows the timing of the light flash and flash intensities are indicated in [log (cd*s/m^2^)]. **E**: B-wave amplitudes of scotopic (SC) and photopic (PH) single flash ERG recordings in *Cdc42* knockdown (red line, n=3) and control (black line, n=4) mice are blotted as a function of the logarithm of flash intensity. Boxes indicate the 25% and 75% quantile range, whiskers the 5% and 95% quantiles and solid lines connect the medians of the data. Scale bars: 50 mm. OS: photoreceptor outer segments. IS: photoreceptor inner segments. ONL: outer nuclear layer. INL: inner nuclear layer. GCL: ganglion cell layer.

Recent studies have shown that CDC42 is involved in photoreceptor morphogenesis and polarity in *Drosophila* [31,32]. To test whether rod-specific ablation of CDC42 has an effect in the mouse retina, we analyzed tissue morphology, the expression of photoreceptor markers, and the retinal function in knockdown animals. The overall retinal morphology and photoreceptor structure of *Cdc42* knockdown mice was maintained relative to controls (Figure 4A). Similarly, ablating CDC42 did not change expression levels of markers for rod and cone photoreceptors (Figure 4B). Furthermore, the intracellular localization pattern of rhodopsin, rod transducin, and short-wavelength (SWL) cone opsin was normal in *Cdc42* knockdowns and indistinguishable from control littermates (Figure 4C). In particular, no mislocalization of these proteins was observed, suggesting that protein transport through the cilium was not affected by the lack of CDC42 (Figure 4C). Normal rod (and retinal) physiology in *Cdc42* knockdown mice was also supported by normal scotopic and photopic ERG responses to light stimuli (Figure 4D). Together, these results suggest that deficiency of CDC42 in adult mouse rods does not influence photoreceptor physiology or function.

### Rod-specific knock-down of CDC42 does not affect progression of photoreceptor degeneration

**Figure 5:**
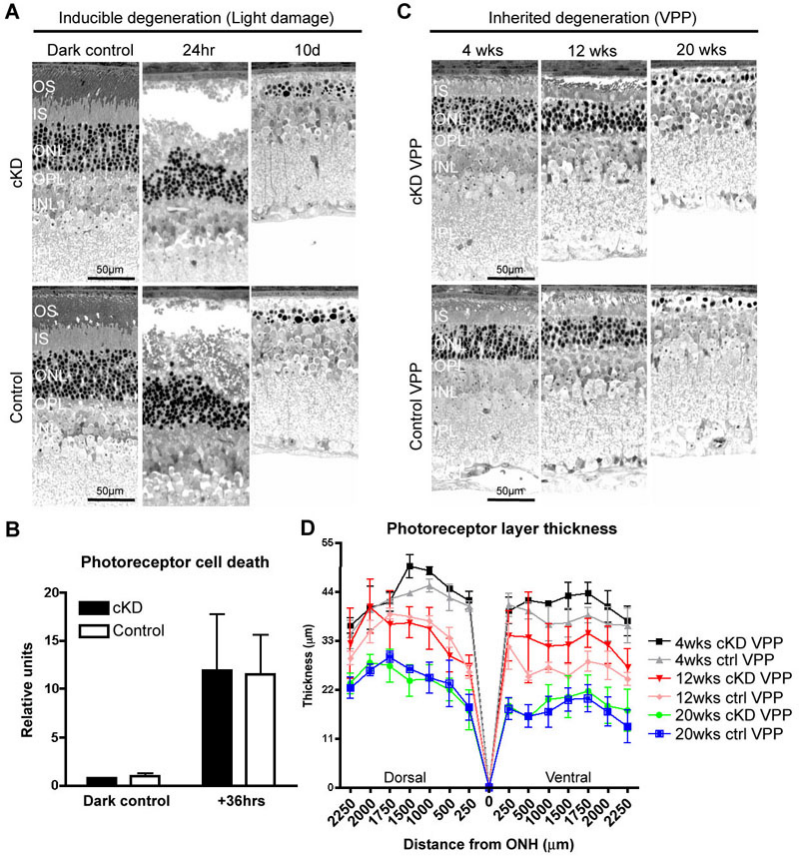
Ablation of cell division cycle 42 homolog (S.cerevisiae; CDC42) does not affect progression of photoreceptor degeneration. **A**: Retinal morphologies of *Cdc42* conditional knockdown (cKD) and control mice before (dark control) or at 24 h and 10 days after light exposure are shown. **B**: Photoreceptor apoptosis was quantified in *Cdc42* knockdown (black bars) and wild-type controls (white bars) 36 h after light exposure. Shown are results from n=4 retinas per time point and genotype. Statistical analysis showed no significant difference in apoptosis between wild-type and knockdown mice (Student *t*-test). **C**: Retinal morphologies of *Cdc42^flox/flox^*;opsin-Cre; VPP (cKD VPP) and *Cdc42^flox/flox^*;VPP (control VPP) mice were examined at 4 weeks (weeks), 12 weeks and 20 weeks of age. Shown are representative sections of n=3. **D**: Thickness of the outer nuclear layer (ONL) of *Cdc42^flox/flox^*;opsin-cre;VPP (cKD) and *Cdc42^flox/flox^*;VPP (ctrl) mice at 4, 12, and 20 weeks of age is shown in a spider diagram. Measurements from morphological sections of 3 mice per genotype and age are shown. Retinal thickness of wild-type and knockdown mice were comparable at all time points and locations (ANOVA [ANOVA] followed by bonferroni post-hoc test). OPL: outer plexiform layer, INL: inner nuclear layer, IPL; inner plexiform layer, GCL: ganglion cell layer, IS: photoreceptor inner segment, OS: photoreceptor outer segment. Scale bars: 50 mm.

RAC1, another classical member of the small Rho GTPases, has recently been shown to have a pro-apoptotic role in light-induced retinal degeneration [21]. The perinuclear localization of CDC42 in TUNEL-negative photoreceptor cells after excessive light exposure (Figure 1 and Figure 2) suggested that CDC42 might have a similar role to RAC1 in degeneration. However, CDC42 ablation did not result in any detectable differences in the progression of induced (light damage) or inherited (VPP) photoreceptor apoptosis (Figure 5). At 24 h after light exposure, retinal morphology showed severely disturbed rod inner and outer segments, as well as a large number of photoreceptor nuclei with condensed chromatin in both control and knockdown mice (Figure 5A). At 10 days after exposure, the ONL was reduced to 2–3 rows of mainly pyknotic photoreceptor nuclei. Biochemical quantification of photoreceptor apoptosis at 36 h after light exposure confirmed that *Cdc42* knockdown mice were susceptible to light injury in a similarly fashion to controls (Figure 5B). Similarly, VPP mice showed a comparable progression of photoreceptor loss independently of the presence or absence of CDC42. At 20 weeks of age, both control and knockdown mice possessed one row of photoreceptor nuclei, most of which contained condensed chromatin (Figure 5C). A similar progression of the degeneration was further supported by the comparable reduction of the ONL thickness at 4, 12, and 20 weeks of age in both control and *Cdc42* knockdown mice (Figure 5D). Therefore, the presence or absence of CDC42 did not influence the susceptibility of rods to degeneration or the progression toward cell death.

### Rod-specific ablation of CDC42 does not affect the induction of endogenous survival pathways after light exposure

**Figure 6:**
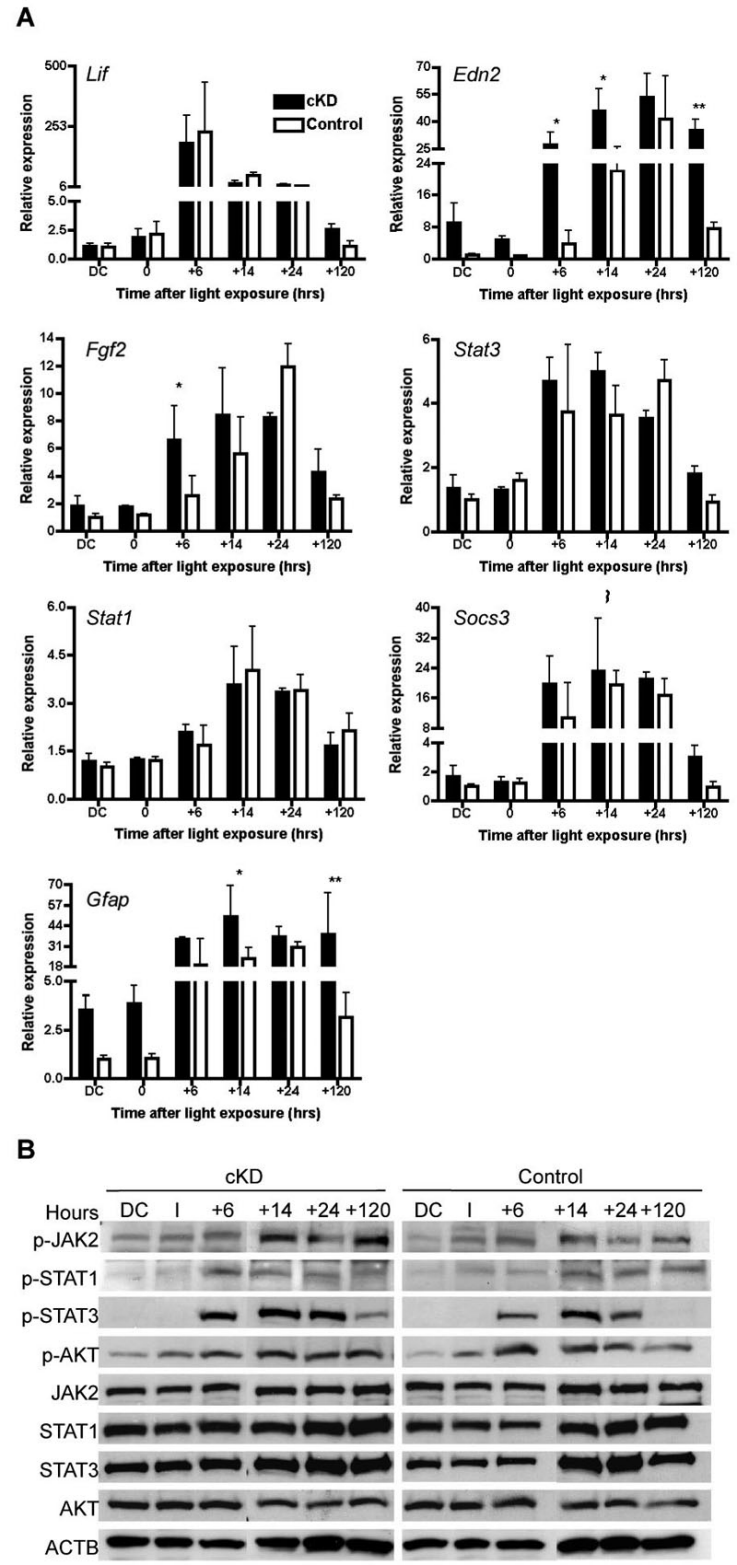
Ablation of cell division cycle 42 homolog (S.cerevisiae; CDC42) does not affect endogenous signaling pathways after light exposure. **A**: Twelve-week-old *Cdc42* conditional knockdown (cKD; black box) and control (white box) mice were or were not (dark control; DC) exposed to 13 klux of white light for 2 h, and retinal tissue was isolated at different time points after light exposure as indicated (0–120 h). Relative mRNA levels of indicated genes were compared to the respective levels of non-exposed control mice (DC), which were set to ‘1’. Shown are mean values±SD of 3 independent mice. Statistical differences of means were calculated using ANOVA (ANOVA) followed by a bonferroni post-hoc test. * p<0.05; ** p<0.01; *** p<0.001. **B**: Total retinal extracts from *Cdc42* cKD and wild type controls (control) at indicated time points after light exposure (as in **A**) were immunoblotted. Shown are representative blots of n=3.

Degeneration of photoreceptors activates a LIF-controlled protective signaling cascade in the retina [33]. This signaling involves induced expression of endothelin 2 (*Edn2*) and fibroblast growth factor 2 (*Fgf2*), as well as activation of JAK2, STAT1, and STAT3 proteins through phosphorylation [33-36]. Since it has been shown in other systems that CDC42 can participate in the endothelin [37] and LIF signaling pathways [38], we tested whether the ablation of CDC42 in rods might affect the molecular response during retinal degeneration. Semiquantitative analysis of gene expression after light exposure showed a similar fold induction over the respective basal levels in control and *Cdc42* knockdown mice (Figure 6A). However, basal expression of *Edn2* and *Gfap* was elevated in retinas of untreated knockdown mice, as well as in the early degeneration phase after light exposure (Figure 6A). Activation of JAK2, STAT1, STAT3, and AKT by phosphorylation was not grossly affected by the lack of CDC42 (Figure 6B). Together, these results suggest that lack of CDC42 in photoreceptors does not strongly affect the regulation of the LIF-controlled survival pathway and corroborates our conclusion of a nonessential role of CDC42 in photoreceptor degeneration.

## Discussion

The small GTPase RAC1 was recently shown to be a pro-apoptotic factor in the model of light-induced retinal degeneration [21]. Here, we show that CDC42, another member of the small GTPase family, does not play a major role in photoreceptor death, even though CDC42 specifically localized to the perinuclear region of some photoreceptor cells during induced and inherited retinal degeneration. Although CDC42 is involved in the maintenance of cellular polarity [19,39] and vesicular trafficking [40], both of which may be important for correct protein localization, ablation of CDC42 did not affect the expression and localization of rod and cone markers. In addition, it was also found that lack of CDC42 also did not affect the dark-adapted levels of rhodopsin and light-driven translocation of transducin in rods (data not shown). Together with the normal ERG of *Cdc42* knockdown mice, our results thus indicate that CDC42 is not necessary for the normal structure and function of mature mammalian photoreceptors. Therefore, CDC42 may either have a nonessential function in mature rods or other small GTPases like the four CDC42-like GTPases might compensate for the lack of CDC42, as suggested by others [41].

CDC42 is not only expressed in photoreceptors but in all layers of the adult retina (Figure 1) [20,42]. In retinal ganglion cells, CDC42 may have modulatory functions and is involved in neurite outgrowth and growth cone dynamics [43,44]. The function of CDC42 in the INL has not been addressed, but the strong immunolabeling in the inner plexiform layer (IPL) and INL may point to a modulatory function in normal neuron physiology rather than neuroprotection. To resolve the function of CDC42 in the inner retina, additional cell-type-specific knockdowns may be needed and analyzed.

Laser scanning confocal microscopy showed that CDC42 localized around the nucleus of some photoreceptors in the degenerating retina (Figure 1 and data not shown). This localization was an early and transient event detected exclusively in TUNEL-negative cells. However, it seems unlikely that this accumulation predisposes cells to survive, since in the rd1 mouse for example, all photoreceptors eventually die despite the detection of perinuclear CDC42 localization early during the degenerative process.

Perinuclear localization of CDC42 has also been found in mammalian cells in vitro during serum-dependent processes [45] and in migrating cortical neurons during development [46]. It has also been reported that CDC42 may be involved in the subcellular distribution of its effector proteins including the mixed lineage kinase 3 [47] and the p21 activated kinase 5 (PAK5) [48]. Interestingly, members of both the mixed lineage kinase and PAK family of proteins have been connected to cell death signaling [49,50]. Altered Pak5 nucleocytoplasmic shuttling, for example, changed the sensitivity of neuroblastoma cells and neural stem cells to apoptosis [48]. However, we did not observe altered intracellular distribution of PAK5 in the degenerating retina (data not shown), suggesting that a CDC42-dependent PAK5 translocation mechanism similar to neuroblastoma may not be involved in photoreceptor apoptosis. Thus, the functional consequences of the perinuclear accumulation of CDC42 in photoreceptors of the degenerating retina need to be further studied.

The perinuclear localization of CDC42 in TUNEL-negative photoreceptor cells during retinal degeneration seems to be a general reaction of the retina to degeneration. This localization points to a prominent role of CDC42 in the retinal response to photoreceptor injury. Several studies have indicated an influence of CDC42 on apoptosis and/or survival through a variety of pathways in different systems [22,51,52]. Many of these publications also include data on RAC1 and suggest overlapping roles of the two GTPases [53,54]. Such an overlap or redundancy can also be suspected for the maintenance of the integrity of normal rod photoreceptors in the adult mouse retina. Separate ablation of RAC1 [21] and CDC42 (this work) did not affect the structure and function of these sensory cells. During photoreceptor degeneration, however, RAC1 and CDC42 may have distinct roles. First, CDC42 localized in TUNEL-negative (Figure 2), whereas RAC1 colocalized with TUNEL-positive cells [55]. Second, the rod-specific knockdown of CDC42 did not prevent photoreceptor loss in the light-induced or inherited model of retinal degeneration—in contrast to the rod-specific ablation of RAC1, which protected rods after excessive light exposure [21]. This protection was attributed to reduced oxidative stress through inhibition of NADPH oxidase [21]. These differences are remarkable and add to the growing list of cellular settings where CDC42 and RAC1 exhibit functional differences.

Our gene expression data showed that light exposure caused a similar fold induction (over basal levels) of genes involved in the LIF-controlled survival pathway in both wild-type and *Cdc42* knockdowns. Similar observations were made for the RAC1 knockdowns, where it was found that the expression of members of LIF pathway was also unaltered [21]. However, the CDC42 knockdown resulted in an increased basal expression of *Gfap* and *Edn2*. Recently, the binding of endothelins to endothelin receptor type beta (EDNRB) and EDNRA was shown to activate astrocytes near the optic nerve head [56]. Indeed, *Cdc42^flox/flox^;*opsin-cre knockdown mice may have an increased astrocytic network in the GCL (data not shown). Astrocyte activation was also reported after *Cdc42* ablation in cortical progenitors during development [57]. The relevance of these observations needs to be elucidated, and it needs to be established how the lack of CDC42 may stimulate *Edn2* expression.

In summary, we showed that CDC42 accumulates in the perinuclear region of photoreceptors during retinal degeneration. However, the susceptibility of photoreceptors to degeneration and progression of cell death were not affected by the *Cdc42* knockdown. Therefore, the precise role of CDC42 in photoreceptors remains to be identified but it may include a modulatory function of the cellular physiology to adapt to stress situations. Our results are especially of relevance when choosing a particular member of the family of small GTPases as a potential target for therapeutic interventions in retinal degenerative diseases.
